# Transgenerational adverse effects of valproate can’t be by-passed

**DOI:** 10.1017/S1092852925000288

**Published:** 2025-06-25

**Authors:** Alain Braillon

**Keywords:** Teratogenicity, neurodevelopmental disorders, autism, epigenetics

The review literature documenting valproic acid associated with anatomical, behavioral, and cognitive teratogenicity^1^ by-passed a 2022 publication of a series of 108 individuals (85 women and 23 men) suffering complications due to valproate exposure in utero who were parents themselves.^2^ Among their 187 children, 88 (47%) had neither malformation nor developmental disorders, 43 had malformation(s) (23%), and 82 (44%) had neurodevelopmental disorders.^2^

This publication, by a patient’s advocate being the mother of two children who were consecutively damaged by valproate, cannot be a surprise. Valproate, a histone deacetylase inhibitor, renders chromatin more transcriptionally active and induces epigenetic changes.^3^ I’m afraid that the concept of translational medicine coined by Cavero in 2007, as the successful migration of preclinical data into predictive models for clinical outcomes, remains an empty concept when the issue is drug safety.

When will there be a concern for adequate information of valproate’s victims about fertility options, antenatal diagnosis, and adequate early surveillance? Of note, the first red flag about valproate, the first of a long series, was published in 1982^4^ but no Risk Minimization Measures before the mid 2010s.
